# Uncovering ancient transcription systems with a novel evolutionary indicator

**DOI:** 10.1038/srep27922

**Published:** 2016-06-16

**Authors:** Naruhiko Adachi, Toshiya Senda, Masami Horikoshi

**Affiliations:** 1 Structural Biology Research Center, Photon Factory, Institute of Materials Structure Science, High Energy Accelerator Research Organization (KEK), 1-1 Oho, Tsukuba, Ibaraki 305-0801, Japan; 2 Precursory Research for Embryonic Science and Technology, Japan Science and Technology Agency, 1-1 Oho, Tsukuba, Ibaraki 305-0801, Japan; 3 Department of Materials Structure Science, School of High Energy Accelerator Science, The Graduate University of Advanced Studies (Soken-dai), 1-1 Oho, Tsukuba, Ibaraki 305-0801, Japan; 4 Laboratory of Developmental Biology, Institute of Molecular and Cellular Biosciences, The University of Tokyo, 1-1-1 Yayoi, Bunkyo-ku, Tokyo 113-0032, Japan

## Abstract

TBP and TFIIB are evolutionarily conserved transcription initiation factors in archaea and eukaryotes. Information about their ancestral genes would be expected to provide insight into the origin of the RNA polymerase II-type transcription apparatus. In obtaining such information, the nucleotide sequences of current genes of both archaea and eukaryotes should be included in the analysis. However, the present methods of evolutionary analysis require that a subset of the genes should be excluded as an outer group. To overcome this limitation, we propose an innovative concept for evolutionary analysis that does not require an outer group. This approach utilizes the similarity in intramolecular direct repeats present in TBP and TFIIB as an evolutionary measure revealing the degree of similarity between the present offspring genes and their ancestors. Information on the properties of the ancestors and the order of emergence of TBP and TFIIB was also revealed. These findings imply that, for evolutionarily early transcription systems billions of years ago, interaction of RNA polymerase II with transcription initiation factors and the regulation of its enzymatic activity was required prior to the accurate positioning of the enzyme. Our approach provides a new way to discuss mechanistic and system evolution in a quantitative manner.

Transcription, which inheres in all living organisms, is a basic mechanism for maintaining and changing cellular states by switching the expression of various genes on and off. Eukaryotes, which contain an RNA polymerase II (pol II) transcription system for messenger RNA synthesis, possess multiple initiation factors called general transcription factors (GTFs)^1^. TBP is a GTF that binds to an upstream sequence of a target gene, the so-called TATA-box, after which another GTF, TFIIB, binds to TBP and its adjacent sequence, the B recognition element (BRE)^2^,^3^,^4^. The TBP-TFIIB complex on the promoter region recruits pol II and additional GTFs, such as TFIIF, TFIIE, and TFIIH^4^. Since TBP and TFIIB/TFIIB-like factor are present in all three eukaryotic RNA polymerase systems (TBP and Rrn7 in the pol I system, TBP and TFIIB in the pol II system, and TBP and Brf1 in the pol III system)^5^ and conserved among archaea and eukaryotes^6^,^7^, the common ancestors of archaea and eukaryotes must also possess TBP and TFIIB in their transcription apparatus. Therefore, elucidation of their molecular evolution should provide critical information about the initial form of the pol II-type transcription apparatus. Moreover, the evolutionary analysis of transcription initiation factors and transcription systems would shed light on the initial forms of cellular life and its evolution.

Although previous studies have reported that the DNA-binding domain of TBP exhibits sequence similarity to that of eubacterial transcription initiation factors (sigma factors)^8^, their tertiary structures were later shown to be different^9^,^10^, suggesting that the two domains have distinct origins. TFIIB was then suggested to have functional similarity to the sigma factors^11^, but their amino acid sequences and tertiary structures were different^11^. So far, therefore, the evolutionary origins of TBP and TFIIB remain elusive. In general, to elucidate the evolution of a particular gene family, a phylogenetic analysis is utilized to estimate the evolutionary distances from the most recent common ancestor (MRCA) to present offspring genes in the gene family^12^ (Fig. 1A,B). First, in the evolutionary analysis, the evolutionary distance for every pair of sequences is calculated (Fig. 1A). Then the calculated evolutionary distances are used to prepare a phylogenetic tree (Fig. 1B). Since all sequences are equivalent on the phylogenetic tree, it is impossible to choose the MRCA among nodes in the phylogenetic tree. There are no reasonable measures for selecting one specific node as the MRCA at this point. However, once we define an outer group, which is usually an isolated clade in the phylogenetic tree, the node nearest to the outer group can reasonably be considered the MRCA of the remaining genes. For example, if the clade containing *Mj (Sa, Pw*, and *Mj*) is chosen as an outer group (the left panel of Fig. 1C), the MRCA of *Sc, At*, and *Hs* can be determined (the black spot in the left panel of Fig. 1C). When the clade containing *Sc (At, Hs*, and *Sc*) is chosen as an outer group (the right panel of Fig. 1C), the MRCA of *Sa, Mj*, and *Pw* can be determined on the phylogenetic tree (the black spot in the right panel of Fig. 1C). In this way, the phylogenetic analysis suggests the evolutionary changes of the family genes. However, a problem arises from this approach^13^: it is impossible to estimate evolutionary distances from the MRCA to all family genes, because some of the family members must be excluded from consideration as an outer group. To obtain the MRCA information for both archaea and eukaryotes, outer group gene information such as eubacterial counterparts is required. To date, however, no TBP counterparts have been identified in eubacteria. Moreover, even if eubacterial counterparts were discovered, we would still need to utilize these counterparts as an outer group. In any case, it is impossible to obtain MRCA information for all known TBP genes. This is an unsolved dilemma in phylogenetic analysis. In order to overcome this limitation, there is need of a novel indicator that circumvents the requirement of an outer group.

Here, we present a new analytical method to estimate evolutionary distances from a common earliest ancestral-gene (EA-gene) to all present offspring genes without using an outer group for the evolutionary analysis of TBP and TFIIB. Our analysis utilizes direct repeat sequences found in TBP and TFIIB as an evolutionary measure of the degree of similarity between the present offspring genes and their ancestors billions of years ago. Considering that the gene duplication is the beginning of a gene with a direct repeat, the evolutionary distance between the first and second repeats can be used as a new indicator of the evolutionary distance between ancestral and present offspring genes. Using this indicator, our analysis suggests the evolutionary changes of TBP and TFIIB. We also provide the first data on the evolutionary development of the transcription apparatus using this indicator.

## Results

### A new indicator for determining the distance from an ancestor

As described in the Introduction, a new indicator is needed to measure the evolutionary distances between an ancestral gene and the whole set of present offspring genes of a particular gene family. Since methods utilizing an outer group cannot overcome the limitation of the phylogenetic analysis, any potential indicators must utilize information embedded in gene sequences as an evolutionary measure. TBP and TFIIB are found in archaea and eukaryotes, and no counterparts have been found in eubacteria.

**Figure 1:**
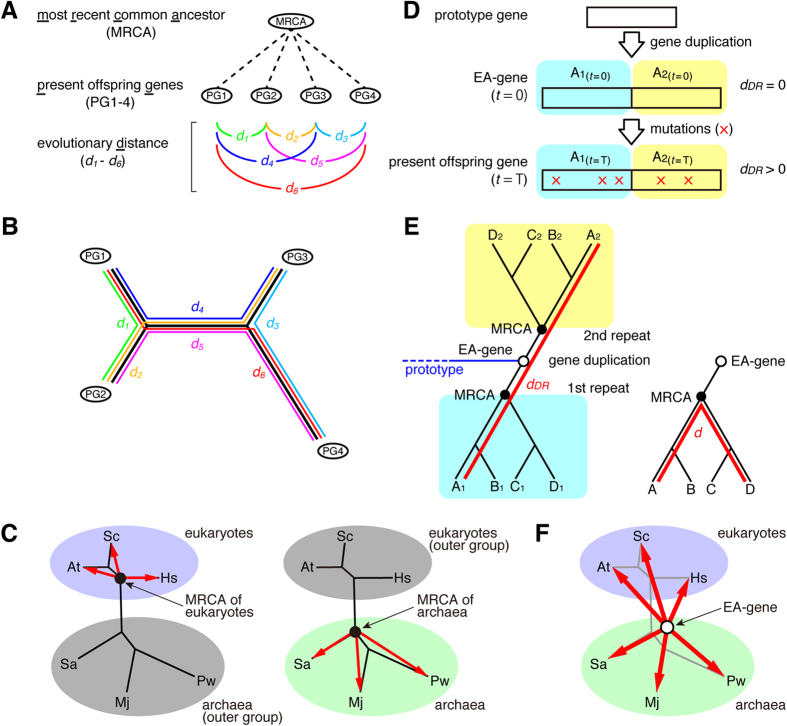
Schematic representation of the present evolutionary distance (*d*) and the distance between the first and second repeats (*d*_*DR*_). (**A**) Schematic representation of evolutionary distances. The evolutionary distances between two genes in the gene family are shown as *d*_1_–*d*_6_. (**B**) Calculated evolutionary distances *d*_1_–*d*_6_ are utilized to prepare an unrooted phylogenetic tree. (**C**) Schematic drawing of an unrooted phylogenetic tree of archaeal and eukaryotic genes. The position of the MRCA for both archaeal and eukaryotic genes cannot be determined on the unrooted phylogenetic tree. When archaeal genes are considered as an outer group (distal relative genes), the MRCA for eukaryotic genes can be placed (left panel). When eukaryotic genes are considered as an outer group, the MRCA for archaeal genes can be placed (right panel). (**D**) Relationship of gene duplication, accumulated mutations, and *d*_*DR*_. The EA-gene (middle panel) is generated by a gene duplication of a prototype gene (upper panel). The *d*_*DR*_ value of the EA-gene (*t* = 0) is zero due to two identical nucleotide sequences in the direct repeat. The *d*_*DR*_ value of the present offspring gene (lower panel) can be utilized as an indicator of the evolutionary distance between the EA-gene and the present offspring gene. (**E**) Relationship between the phylogenetic tree and *d*_*DR*_ defined in this study. *d* is the path length “between two distinct genes” *via* their MRCA (*e.g.*, the red line in the right panel)^12^. On the other hand, *d*_*DR*_ is the path length “between the first and second repeats in one gene” *via* the hypothetical EA-gene (*e.g.*, the red line in the left panel). Therefore, *d* reflects the evolutionary distance between the present gene and one of the ancestral genes, but *d*_*DR*_ could be a reasonable indicator of the evolutionary distance between a present gene and its EA-gene. (**F**) Schematic drawing of the relationship between the phylogenetic tree and *d*_*DR*_. The usual phylogenetic tree is prepared based on the evolutionary distances (*d*) of two genes in the gene family. On the other hand, *d*_*DR*_ is a reasonable indicator of the evolutionary distances between the EA-gene and each of the present offspring genes.

We noticed that information in the direct repeat sequences present in both TBP and TFIIB could be utilized for analyzing the evolution of the genes without an outer group. Generation of a direct repeat can be considered the starting point of the molecular evolution of the direct repeat-containing genes. It should be noted that the first and second repeats are identical at the time of its generation (middle panel in Fig. 1D). In this study, we designate a direct repeat-containing gene that was generated just after gene duplication as the earliest ancestral-gene (EA-gene) in order to distinguish the EA-gene from other types of ancestral genes, including the MRCA. The EA-gene can be considered the first-appearing common ancestor of a direct repeat-containing gene.

Importantly, the nucleotide sequences of the first and second repeats, which were once the same, have diverged by mutations during evolution. Therefore, a sequence comparison between the first and second repeats allows us to evaluate the evolutionary distance between the EA-gene and its offspring gene(s) (lower panel in Fig. 1D). Here, we would like to propose that the evolutionary distance between the first and the second repeats can be utilized as an indicator of the evolutionary distance between the EA-gene and its offspring gene(s); the newly defined evolutionary indicator is designated as *distance between Direct Repeat (d*_*DR*_). Obviously, *d*_*DR*_ is zero (*d*_*DR*_ = 0) for the EA-gene, because nucleotide sequences of the first and second repeats were identical at that time. Thereafter, *d*_*DR*_ gradually increased due to accumulated mutations in the course of evolution.

We can now analyze the molecular evolution using *d*_*DR*_, and we designate this novel method the “*d*_*DR*_ analysis”. It must be noted that the new indicator *d*_*DR*_ is essentially different from the general evolutionary distance (*d*) (Fig. 1E). The *d*_*DR*_ analysis does not utilize an outer group and thus *d*_*DR*_ could be a reasonable indicator of the evolutionary distance between a present offspring gene and its EA-gene (Fig. 1F).

### Two criteria for the new indicator

The appropriate calculation of *d*_*DR*_ needs to satisfy two criteria. First, gene duplication of the target gene should occur only once during the evolution of family genes. If an offspring gene has a second gene duplication, the accumulated mutations of the offspring gene will be lost at that time point; in such a case it can be considered that another EA-gene is generated among the family genes. When comparing *d*_*DR*_, the EA-gene should be the same among the target genes, because the EA-gene is defined as the origin of the evolution of the family genes. Therefore, we must examine whether or not the gene duplication of a target gene has occurred only once.

The method reported by Gogarten *et al*.^14^ and Iwabe *et al*.^15^ can be used to make this determination. In the case of direct repeat genes, a phylogenetic tree is prepared by using the first and second repeats of the target genes. The branch pattern of the obtained phylogenetic tree provides information on gene duplication in the evolution of the target genes^6^. A phylogenetic tree of genes generated by single gene duplication shows two distinct clades that consist of the first and second repeats, respectively. If gene duplication occurred more than once, each clade in the phylogenetic tree would consist of a mixture of the first and second repeats.

The second criterion is that the direct repeats in the target genes should have a moderate conservation ratio. Our indicator cannot be applied to genes that show a low conservation ratio between the first and second repeats. Since nucleotide sequences are composed of four nucleotides, a minimum sequence identity of approximately 25% is permitted between two random nucleotide sequences, if there is no bias in nucleotide substitutions in the evolution. Therefore, we cannot distinguish between random noise and significant homology of the nucleotide sequences in the direct repeats, when the conservation ratio of the direct repeats is close to 25%.

### Evolutionary distances to the EA-gene of TBP

**Figure 2:**
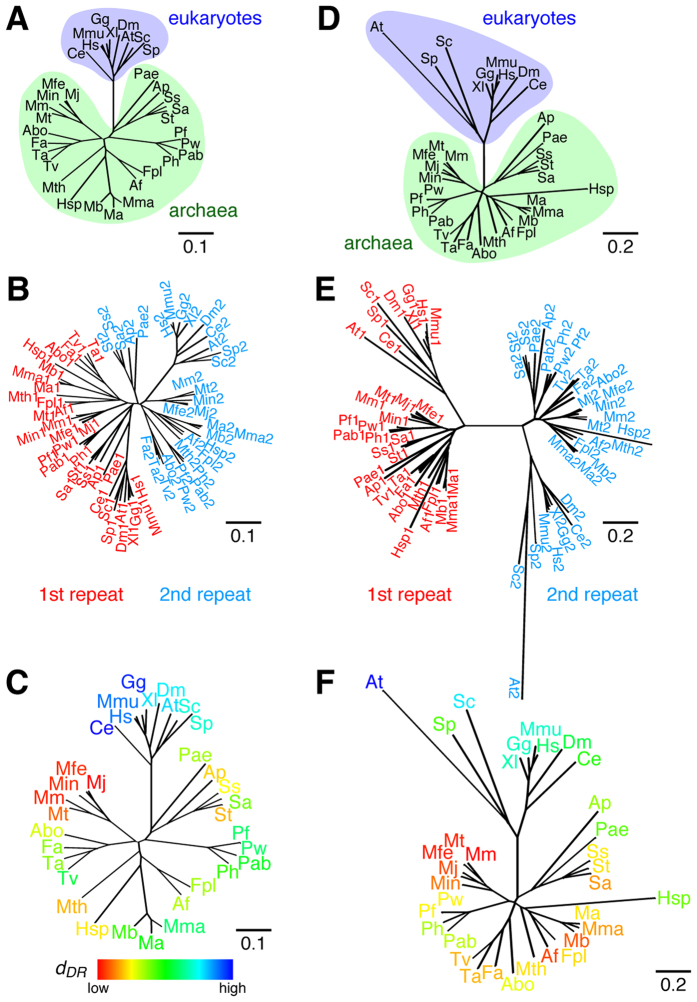
Direct repeats present within TBP and TFIIB are derived from their EA-genes generated by single gene duplication. (**A**,**D**) Phylogenetic trees drawn with the nucleotide sequences of the conserved core region of TBP (**A**) and TFIIB (**D**) from 34 species. Abbreviations of species names are given in the footnote of Table 1. (**B**,**E**) Phylogenetic trees drawn with the nucleotide sequences of the first and second repeats of TBP (**B**) and TFIIB (**E**) from 34 species. Red and cyan indicate, respectively, the first and second repeats of the TBP (**B**) and TFIIB (**E**) genes. (**C**,**F**) The *d*_*DR*_ values of TBP (**C**) and TFIIB (**F**) are shown on their phylogenetic trees using red-blue coloring by the *d*_*DR*_ values.

Prior to calculation of the *d*_*DR*_ values for TBP genes, we examined whether or not TBP genes satisfied the two aforementioned criteria. TBP has a direct repeat with approximately 180 amino acids in the C-terminal core region^8^,^16^. Accordingly, the tertiary structure of the corresponding region of TBP has a pseudo-two-fold axis^9^. Therefore, the EA-gene of TBP seems to be generated by gene duplication of a prototype gene. We examined the nucleotide sequences of TBP from 34 species (Fig. 2A) and found that the first and second repeats of the TBP genes were clearly divided into two distinct clades (Fig. 2B), indicating that the first criterion for our analysis was satisfied for the TBP genes.

Next, we examined whether the 34 TBP genes satisfied the second criterion. The first and second repeats of the 34 TBP genes were compared on the basis of a nucleotide sequence alignment (Supplementary Fig. S1). Since the sequence identities between the first and second repeats of these TBP genes ranged from 39.9% to 61.5%, the second criterion for our analysis was also satisfied (Table 1A).

Since all 34 TBP genes satisfied the two criteria, the *d*_*DR*_ value for each TBP gene was calculated (Table 1A). Since the *d*_*DR*_ values represent the distances from the EA-gene, all of the present TBP genes could be ordered according to their similarities to the EA-gene. The results also showed that archaeal TBP genes have *d*_*DR*_ values (0.488–0.933) lower than those of eukaryotic genes (1.00–1.22) (Table 1A). Among all the examined TBP sequences, the TBP gene from *M. jannaschii* (hereafter *Mj* TBP) exhibited the lowest *d*_*DR*_ (0.488). Species that are branched into the same clade of *M. jannaschii (Methanococcus maripaludis, Methanocaldococcus fervens, Methanocaldococcus infernus*, and *Methanothermococcus thermolithotrophicus*) also have TBP genes with low *d*_*DR*_ values (0.516, 0.524, 0.524 and 0.560, respectively) (Fig. 2C). These results indicate that *Mj* TBP and its close relative genes are more similar to the EA-gene than to the other genes.

### Evolutionary distances to the EA-gene of TFIIB

The molecular evolution of TFIIB was also analyzed according to the *d*_*DR*_ values. TFIIB contains a direct repeat with approximately 190 amino acids in the C-terminal core region^17^,^18^. Accordingly, the tertiary structure of the corresponding region of TFIIB has a tandem cyclin fold^19^,^20^ (Supplementary Fig. S2). The same analysis^14^,^15^ for TFIIB genes in the present 34 species (Fig. 2D) indicated that the first and second repeats of the TFIIB genes are clearly divided into two distinct clades (Fig. 2E), and thus that the first criterion for our analysis was satisfied for the TFIIB genes. Since the sequence identities between the first and second repeats of these TFIIB genes ranged from 32.6% to 51.4%, the second criterion for the *d*_*DR*_ based analysis was also satisfied (Table 1B).

The calculation of the *d*_*DR*_ values for the TFIIB genes also allowed the present TFIIB genes to be ordered according to their similarities to the EA-gene, revealing that archaeal TFIIB genes have low *d*_*DR*_ (0.784–1.26) and eukaryotic ones have high *d*_*DR*_ (1.28–1.95) (Table 1B), as observed in the case of the TBP genes (Table 1A). Among these genes, the TFIIB gene from *M. maripaludis* (hereafter *Mm*TFIIB) showed the smallest *d*_*DR*_ among the 34 TFIIB genes (0.784). *M. maripaludis* and *M. jannaschii* are branched into the same clade and, like TBP, species in this clade (*M. fervens, M. thermolithotrophicus, M. jannaschii, M. infernus*) have TFIIB genes with low *d*_*DR*_ values (0.820, 0.828, 0.869 and 0.918, respectively) (Fig. 2F).

Introduction of a new evolutionary indicator, *d*_*DR*_, thus enabled us to estimate the evolutionary distances between the EA-gene and present offspring genes for TBP and TFIIB without setting an outer group. Moreover, the combination of *d*_*DR*_ and a phylogenetic tree allowed us to determine the clade containing genes most similar to the EA-gene at a glance (Fig. 2C,F).

### Analysis of evolutionary change of the amino-acid composition

**Figure 3:**
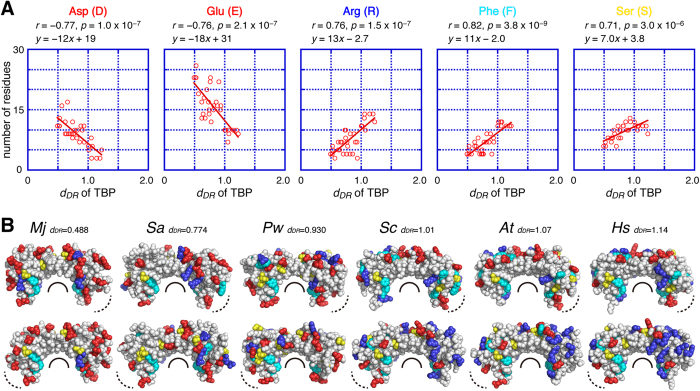
Correlation between *d*_*DR*_ and the amino-acid compositions of TBP. (**A**) Correlations between *d*_*DR*_ and the number of the specific amino-acid residues for TBP (Asp (**D**), Glu (**E**), Arg (R), Phe (**F**), and Ser (S)). The best fitting lines are shown in red. The correlation coefficient (r) and p-value (p) are shown in each graph. (**B**) Sphere models of TBP molecules from *M. jannaschii (Mj*), *S. acidocaldarius (Sa*), *P. woesei (Pw*), *S. cerevisiae (Sc*), *A. thaliana (At*), and *H. sapiens (Hs*). The upper and lower panels show front and back views, respectively. Asp and Glu residues are shown in red, Arg residues are shown in blue, Phe residues are shown in cyan, and Ser residues are shown in yellow. The *d*_*DR*_ value of each molecule is also shown. Black curved lines indicate the DNA binding surface of TBP. Dotted curved lines indicate the TFIIB binding surface of TBP.

Since *d*_*DR*_ reflects the evolutionary distance between the EA-gene and its present offspring gene(s), we prepared a list ranking the 34 TBP genes according to their similarities to the EA-gene (Table 1A). This list was utilized to predict evolutionary changes of the amino-acid composition of TBP (Supplementary Table S1A). The *d*_*DR*_ values for the TBP genes show a significant correlation with the numbers of Asp, Glu, Arg, Phe, and Ser residues; the *d*_*DR*_ value and the numbers of Asp and Glu residues have a strong negative correlation (r = −0.77 and −0.76, respectively) and the numbers of Arg, Phe, and Ser residues show a strong positive correlation (r = 0.76, 0.82 and 0.71, respectively) (Fig. 3A).

These results are consistent with the fact that, while the core region of all TBPs has a hydrophobic DNA-binding surface surrounded by positively charged residues, the surface properties of the other core regions show significant difference among species. TBP has a larger number of acidic residues than basic residues in *M. jannaschii* (34 acidic and 22 basic residues)^21^, an almost equal number of both charged residues in *Sulfolobus acidocaldarius* (22 acidic and 26 basic residues)^22^ and *Pyrococcus woesei* (26 acidic and 25 basic residues)^23^, and a smaller number of acidic residues than basic residues in *Saccharomyces cerevisiae* (15 acidic and 27 basic residues)^24^, *Arabidopsis thaliana* (15 acidic and 29 basic residues)^25^, and *Homo sapiens* (13 acidic and 29 basic residues)^26^ (Fig. 3B).

The strong correlation between *d*_*DR*_ and the number of Asp, Glu, Arg, Phe, and Ser residues suggested that the observed differences of amino-acid composition of TBP proteins might be correlated with the evolutionary changes of TBP genes. In addition, assuming that the relationship between *d*_*DR*_ and the amino-acid composition is linear throughout the evolution, we can make another quite informative prediction (see Discussion).

**Figure 4:**
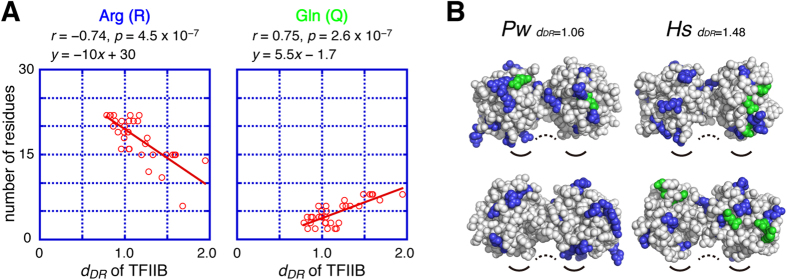
Correlation between *d*_*DR*_ and the amino-acid compositions of TFIIB. (**A**) Correlations between *d*_*DR*_ and the number of the specific amino-acid residues for TFIIB (Arg (R) and Gln (Q)). The best fitting lines are shown in red. The correlation coefficient (r) and p-value (p) are shown in each graph. (**B**) Sphere models of TFIIB molecules from *P. woesei (Pw*) and *H. sapiens (Hs*). The upper and lower panels show front and back views, respectively. Arg residues are shown in blue, and Gln residues are shown in green. The *d*_*DR*_ value of each molecule is also shown. Black curved lines indicate the DNA binding surface of TFIIB. Dotted curved lines indicate the TBP binding surfaces of TFIIB.

In the case of TFIIB, *d*_*DR*_ and evolutionary changes of the amino-acid composition were also compared (Supplementary Table S1B). *d*_*DR*_ and the number of Arg residues have a strong negative correlation with *d*_*DR*_ (r = −0.74), and the number of Gln residues has a strong positive correlation (r = 0.75) (Fig. 4A and Supplementary Table S1B). However, the number of Asp, Glu, Phe, and Ser residues showed no correlations with *d*_*DR*_ in TFIIB. This is consistent with the fact that the surface property of the core region of TFIIB does not show large differences between *P. woesei*^27^ and *H. sapiens*^20^ (Fig. 4B).

### Evolutionary relationship between TBP and TFIIB

**Figure 5:**
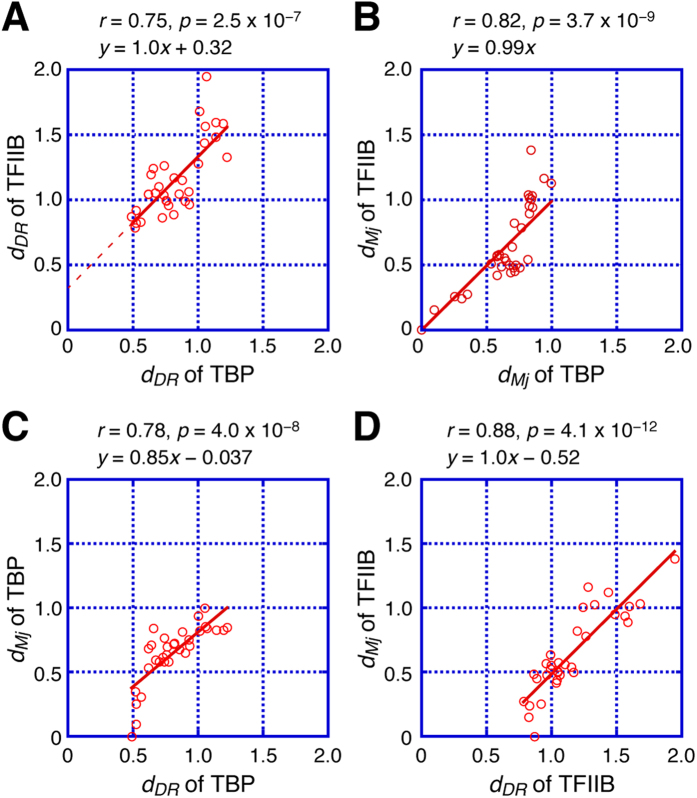
Evolutionary relationship between TBP and TFIIB. (**A**,**B**) Evolutionary correlation between the TBP and TFIIB genes analyzed by *d*_*DR*_ (**A**) and *d*_*Mj*_ (**B**). The best fitting lines are shown in red. The correlation coefficient (r) and p-value (p) are shown. (**C**,**D**) Correlation between *d*_*DR*_ and *d*_*Mj*_ for TBP (**C**) and TFIIB (**D**). The correlation coefficient (r) and p-value (p) for each fitting are shown. Several plots near the x-axis deviate from the best fitting lines. These are plots for close relatives of *M. jannaschii*. Since the starting point of *d*_*Mj*_-calculation is the *M. jannaschii* gene, *d*_*Mj*_ decreases quickly in the close relatives of *M. jannaschii*. However, *d*_*DR*_ does not decrease in the close relatives of *M. jannaschii*, because the starting point of *d*_*DR*_-calculation is the EA-gene. This is the reason for the observed deviations from the best fitting line in the *d*_*Mj*_ plots.

In order to analyze the evolutionary relationship between TBP and TFIIB, their *d*_*DR*_ values were compared. As a result, a significant correlation was found between their *d*_*DR*_ values (r = 0.75, p < 0.01) (Fig. 5A). The coefficient of the best fitting line is approximately 1.0. Assuming that the comparison of the *d*_*DR*_ values between TBP and TFIIB is evolutionary valid, the mutations in the TBP and TFIIB genes seem to have been accumulated at a nearly similar rate when measuring the evolutionary distance with *d*_*DR*_ (Fig. 5A, see Discussion).

We next analyzed the relationship between *d*_*DR*_ and the general evolutionary distance (*d*) to assess the consistency between the two values. In this analysis, evolutionary distances between *M. jannaschii* and each of another species (*d*_*Mj*_) were utilized (Supplementary Table S2), because our *d*_*DR*_ analyses suggested that the TBP and TFIIB genes from *M. jannaschii* were one of the most similar genes to their EA-genes (Table 1).

First, we examined the evolutionary correlation between the TBP and TFIIB genes using the *d*_*Mj*_ values. As shown in Fig. 5B, the *d*_*Mj*_ values of the TBP genes showed a good correlation with those of the TFIIB genes (r = 0.82, p < 0.01), with the coefficient of the best fitting line being approximately 1.0. These are essentially identical to the results obtained using the *d*_*DR*_ values (Fig. 5A). In addition, there was a strong correlation between *d*_*DR*_ and *d*_*Mj*_ in both the TBP and TFIIB genes (r = 0.78, p < 0.01 for TBP; r = 0.88, p < 0.01 for TFIIB) (Fig. 5C,D). These results showed that the analysis using our indicator *d*_*DR*_ was consistent with that using *d*_*Mj*_, suggesting that *d*_*DR*_ can also be utilized as an indicator of the molecular evolution.

It is of note that *d*_*DR*_ and *d*_*Mj*_ are calculated in different ways. The *d*_*Mj*_ value is calculated by using two different genes, and considered as “molecular clock”^28^. On the other hand, the *d*_*DR*_ is calculated by using the first and second repeats of a single gene, and can be designated as a molecular clock inside a gene (*i.e.*, an internal molecular clock). Because the two values are derived from distinct nucleotide-sequence comparisons, it seems unlikely that their strong correlation (0.78 for TBP and 0.88 for TFIIB) is merely coincidental, and thus *d*_*DR*_ would appear to be a reasonable indicator for analyzing molecular evolution.

## Discussion

**Figure 6:**
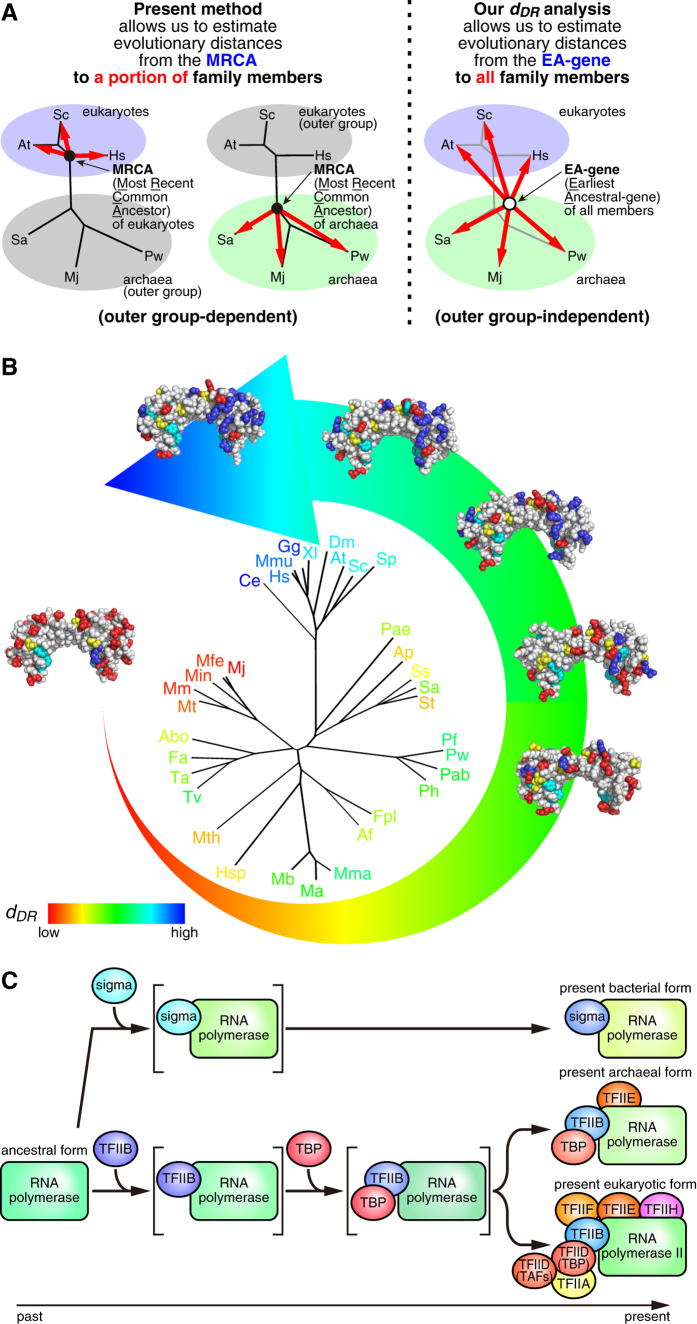
Schematic representation of the *d*_*DR*_ analysis and its application. (**A**) Comparison between the present method (*d*) and our novel analysis (*d*_*DR*_). (**B**) Schematic representation of the evolutionary development of TBP. The *d*_*DR*_ values of TBP are indicated on the phylogenetic trees using a color continuum from red (low *d*_*DR*_) to blue (high *d*_*DR*_). (**C**) Our analysis implies that the TBP gene was generated after the emergence of the TFIIB gene. This is the first time that the emerging order of TBP and TFIIB genes has been reported in the study of molecular evolution, and our results should thus provide novel insights into the evolutionary development of the transcription apparatus and other systems. The order of emergence of other general transcription factors, such as TAFs, TFIIA, TFIIE, TFIIF, TFIIH, remains unknown.

In this work, we introduced a new indicator of molecular evolution, *d*_*DR*_, to estimate the evolutionary distance between the EA-gene and its present offspring gene(s) (Figs 1 and 2, and Supplementary Fig. S3). The *d*_*DR*_ analysis can be applied to any genes which contain an intramolecular direct repeat and satisfy the two aforementioned criteria (see “Two criteria for the new indicator”). Since no outer group is required to calculate the *d*_*DR*_ values, the dilemma described in the Introduction can be overcome (summarized in Fig. 6A). As a result, we were able to obtain novel information about the molecular evolution of TBP and TFIIB present in both archaea and eukaryotes.

First, the *d*_*DR*_ analysis enabled us to predict the species whose TBP and TFIIB genes are most similar to their EA-genes (Fig. 6B, Table 1). In previous studies, the ancestral transcription system has been discussed under the hypothesis that the archaea maintain their early molecular system. This assumption is based on several lines of collateral evidence: for example, the components of the archaeal transcription system are simpler than those of the eukaryotic system. Additionally, the current evolutionary method using eubacterial RNA polymerase (RNAP) as an outer group shows that the subunits of archaeal RNAP are more similar to their MRCA than those of eukaryotic pol II^29^,^30^. In this study, our *d*_*DR*_ analysis has succeeded for the first time in providing direct and quantitative evidence that this hypothesis is reasonable from the viewpoint of the molecular evolution of TBP and TFIIB (Table 1). Our finding is also consistent with a recent study which found that the archaeal root sits within the methanogens^13^.

Second, the *d*_*DR*_ values of the TBP and TFIIB genes from various species can also be utilized to predict the amino-acid composition of their EA-proteins (Figs 3 and 4). Assuming that the relationship between *d*_*DR*_ and the amino-acid composition is linear during evolution and the best fitting line can be extrapolated to the y-axis, the EA-protein of TBP may contain approximately 19 Asp, 31 Glu, no Arg, no Phe, and 4 Ser residues (Fig. 3A), suggesting that the EA-protein of TBP has a more acidic molecular property than that of *Mj* TBP. In the same way, the EA-protein of TFIIB might have a more basic molecular property than that of *Mm*TFIIB (Fig. 4A). It may also be suggested that the number of acidic residues in TBPs and basic residues in TFIIB will decline in the distant future.

Although we previously reported that the surface charge distribution of TBP molecules show significant differences^21^, until now it has been difficult to explain the phenomenon with relation to their molecular evolution. The *d*_*DR*_ analysis provides an explanation. The TBP gene seems to have been generated from its prototype gene by duplication, and the EA-protein of TBP seems to have been an acidic molecule and then to have decreased its acidic residues, probably to improve its interaction(s) with other factors, such as DNA and TBP-interacting proteins. On the other hand, the EA-protein of TFIIB seems to have been a basic molecule and then to have decreased its Arg residues in the course of its evolution, also probably to improve its interaction with other factors, such as TBP, whose Arg residues were increased over the course of its own evolution. These changes in the numbers of Arg residues may have contributed to a reduction in the electrostatic repulsion between TBP and TFIIB.

Interestingly, the number of Phe residues in TBP and that of Gln residues in TFIIB have a positive correlation with *d*_*DR*_. Since these two residues have only two codons, their numbers in a molecule are likely to be decreased by random mutations. These observations suggest that the mutations accumulated on these residues are not random but rather under a specific selective pressure. There might be a mechanism underlying the observed evolutionary tendencies and the relationships of the amino-acid compositions in TBP and TFIIB (Supplementary Table S1). Further analyses may uncover this mechanism.

When the evolutionary development of archaeal and eukaryotic transcription apparatus is considered, the following question is immediately raised: how was the TBP-TFIIB system established? Three possibilities can be considered for the evolutionary emergence of the TBP-TFIIB system: (i) TBP was generated first, (ii) TFIIB was generated first, or (iii) both were generated at the same time (Fig. 6C). The *d*_*DR*_ analysis allows us to envisage the evolutionary development of the archaeal and eukaryotic transcription apparatus. Under the assumptions that the *d*_*DR*_ values of TBP and TFIIB can be compared directly, the relationship between the *d*_*DR*_ values of TBP and TFIIB is linear throughout evolution and the best fitting line can be extrapolated to the y-axis, a surprising result was obtained (Fig. 5). Since the coefficient of the best fitting line in Fig. 5A is approximately 1.0 and mutations in the TBP and TFIIB genes are accumulated at a nearly similar rate (Fig. 5A), the positive y-intercept of the best fitting line implies that mutations had already been accumulated on the TFIIB gene when the TBP gene was generated. This in turn suggests that the TFIIB gene was generated before the TBP gene.

Considering that TFIIB is a pol II interacting-factor and forms a complex with pol II^11^, functional modulation of pol II might be evolutionarily initiated by direct interaction with TFIIB. This is one possible hypothesis to explain the development of the early transcription apparatus and its regulation. It is interesting that the crystal structures of the pol II-TFIIB complex^11^,^29^,^30^ and eubacterial RNAP-sigma complex^10^,^31^,^32^,^33^ suggested a functional relationship between TFIIB and sigma factor. Pol II/RNAP and its interacting factor(s) such as TFIIB and sigma factor would be earlier forms of the transcription apparatus in the evolution, as the *d*_*DR*_ analysis suggested. On the other hand, TBP does not form a stable complex with pol II^11^ but forms a complex with various GTFs for transcriptional activation^34^,^35^,^36^. The ability of TBP to interact with other GTFs may have been acquired in earlier archaea and eukaryotes along with the change of the surface properties of TBP. Since the environment surrounding eubacteria and archaea is known to affect the amino-acid compositions of their molecules^37^, a large difference in the environment between earlier archaea and eukaryotes could have facilitated the change in the surface properties of eukaryotic TBP, leading to the association of other GTFs to establish a more complicated regulatory system of transcription.

This hypothesis could not be proven from the present method(s) of molecular evolution, because by these methods the order of evolutionary emergence of different genes is only estimated from their different distribution among various species^12^. For instance, genes encoding transcription enzymes exist in all three domains, but TBP genes do not exist in eubacteria^6^,^7^, suggesting that the emergence of transcription enzyme genes occurred earlier than the emergence of TBP genes. However, this idea could not be applied to the relationship between TBP and TFIIB, since their distributions among species are the same; both TBP and TFIIB only exist in archaea and eukaryotes. Provided that several conditions can be assumed, the *d*_*DR*_ analysis could predict the order of emergence of the TBP and TFIIB genes for the first time. Further development of a quantitative measurement of molecular evolution would provide detailed insights into the evolutionary development of the transcription apparatus at the system level. We believe that our approach may ultimately lead to a new field of molecular evolution, which might be called “mechanistic and system evolution”.

## Methods

### Sequence analyses

Evolutionary distances (*d*_*DR*_ and *d*) were calculated using the nucleotide sequences of the TBP and TFIIB genes from 34 species (Table 1). All sequences were derived from the NCBI database. Multiple nucleotide-sequence alignments of direct repeats were performed as follows. First, a multiple amino-acid-sequence alignment of the core region was generated with the amino-acid-sequence data of the 34 species (Table 1) by using ClustalW2^38^, and the alignment was manually improved according to the results of BLAST2^39^. Then, the amino-acid sequences of the first and second repeats of TBP and TFIIB were aligned using the amino-acid-sequence alignment of the core region on the basis of the tertiary structural superposition of the first and second repeats of TBP and TFIIB. The tertiary structural superposition was obtained from the DALI software^40^. Then, the nucleotide-sequence alignments were prepared on the basis of the amino-acid-sequence alignments for the first and second repeats of TBP and TFIIB (Supplementary Figs S1 and S2). Evolutionary distances and Newick formats were calculated by the program MEGA5.05 by using a maximum composite likelihood method with default parameters^41^. Nodes of phylogenetic trees in Fig. 2A,C,D and F were swapped by NJplot^42^ according to the *d*_*DR*_ value. Phylogenetic trees were drawn by the program Unrooted^42^. The molecular graphics were prepared by PyMOL (http://www.pymol.org).

## Additional Information

**How to cite this article**: Adachi, N. *et al*. Uncovering ancient transcription systems with a novel evolutionary indicator. *Sci. Rep.* **6**, 27922; doi: 10.1038/srep27922 (2016).

## Supplementary Material

Supplementary Information

## Figures and Tables

**Table 1:** Identity between the first and second repeats and *d*_*DR*_ of the TBP (A) and TFIIB (B) genes.

**(A) TBP**					**(B) TFIIB**				
**species**	**identical bases**	**compared bases**	**identity [%]**	***d***_***DR***_	**species**	**identical bases**	**compared bases**	**identity [%]**	***d***_***DR***_
*Mj*	168	273	61.5	0.488	*Mm*	145	282	51.4	0.784
*Mm*	167	276	60.5	0.516	*Mfe*	144	282	51.1	0.820
*Mfe*	164	273	60.1	0.524	*Mt*	141	282	50.0	0.828
*Min*	164	276	59.4	0.524	*Af*	140	282	49.6	0.862
*Mt*	160	276	58.0	0.560	*Mj*	139	282	49.3	0.869
*Mth*	155	273	56.8	0.619	*Mb*	139	282	49.3	0.886
*St*^†^	157	276	56.9	0.619	*Min*	135	282	47.9	0.918
*Ap*^†^	154	276	55.8	0.639	*Sa*^†^	133	282	47.2	0.957
*Hsp*	156	279	55.9	0.656	*Mma*	133	282	47.2	0.963
*Ss*^†^	152	276	55.1	0.672	*Tv*	128	282	45.4	0.988
*Abo*	143	279	51.3	0.699	*Ta*	130	282	46.1	0.991
*Af*	143	279	51.3	0.726	*Fa*	131	282	46.5	0.993
*Fpl*	138	279	49.5	0.737	*Fpl*	125	282	44.3	1.04
*Pae*^†^	144	276	52.2	0.740	*Mth*	125	282	44.3	1.04
*Fa*	141	279	50.5	0.755	*St*^†^	126	282	44.7	1.04
*Ta*	141	279	50.5	0.759	*Ma*	127	282	45.0	1.04
*Sa*^†^	141	276	51.1	0.774	*Ss*^†^	125	282	44.3	1.05
*Mb*	135	279	48.4	0.814	*Pf*	120	282	42.6	1.06
*Ph*	135	276	48.9	0.816	*Pw*	120	282	42.6	1.06
*Ma*	132	279	47.3	0.855	*Abo*	121	282	42.9	1.10
*Pab*	131	276	47.5	0.879	*Pab*	114	282	40.4	1.15
*Tv*	130	279	46.6	0.902	*Ph*	114	282	40.4	1.17
*Pf*	127	276	46.0	0.930	*Ap*^†^	117	297	39.4	1.19
*Pw*	127	276	46.0	0.930	*Hsp*	118	282	41.8	1.24
*Mma*	127	279	45.5	0.933	*Pae*^†^	113	282	40.1	1.26
*Sp*^‡^	125	273	45.8	1.00	*Sp*^‡^	115	282	40.8	1.28
*Sc*^‡^	121	273	44.3	1.01	*Ce*^‡^	113	282	40.1	1.33
*Dm*^‡^	118	276	42.8	1.05	*Dm*^‡^	108	282	38.3	1.44
*Xl*^‡^	119	276	43.1	1.06	*Hs*^‡^	110	282	39.0	1.48
*At*^‡^	119	279	42.7	1.07	*Xl*^‡^	105	282	37.2	1.56
*Mmu*^‡^	111	276	40.2	1.14	*Gg*^‡^	103	282	36.5	1.59
*Hs*^‡^	113	276	40.9	1.14	*Mmu*^‡^	106	282	37.6	1.59
*Gg*^‡^	110	276	39.9	1.19	*Sc*^‡^	102	282	36.2	1.68
*Ce*^‡^	110	273	40.3	1.22	*At*^‡^	92	282	32.6	1.95

No marks: euryarchaeota; ^†^crenarchaeota; ^‡^eukaryotes.

Species names are abbreviated as follows: *Mj: Methanocaldococcus jannaschii; Mm: Methanococcus maripaludis; Mfe: Methanocaldococcus fervens; Min: Methanocaldococcus infernus; Mt: Methanothermococcus thermolithotrophicus; Mth: Methanothermobacter thermautotrophicus; St: Sulfolobus tokodaii; Ap: Aeropyrum pernix; Hsp: Halobacterium sp* NRC1; *Ss: Sulfolobus solfataricus; Abo: Aciduliprofundum boonei; Af: Archaeoglobus fulgidus; Fpl: Ferroglobus placidus; Pae: Pyrobaculum aerophilum; Fa: Ferroplasma acidarmanus; Ta: Thermoplasma acidophilum; Sa: Sulfolobus acidocaldarius; Mb: Methanosarcina barkeri; Ph: Pyrococcus horikoshii; Ma: Methanosarcina acetivorans; Pab: Pyrococcus abyssi; Tv: Thermoplasma volcanium; Pf: Pyrococcus furiosus; Pw: Pyrococcus woesei; Mma: Methanosarcina mazei; Sp: Schizosaccharomyces pombe; Sc: Saccharomyces cerevisiae; Dm: Drosophila melanogaster; Xl: Xenopus laevis; At: Arabidopsis thaliana; Mmu: Mus musculus; Hs: Homo sapiens; Gg: Gallus gallus; Ce: Caenorhabditis elegans.*
