# Supplementary material for: Uncovering ancient transcription systems with a novel evolutionary indicator
Source: Sci Rep. 2016 Jun 16;6:27922. doi: 10.1038/srep27922 (PMC4910066; doi:10.1038/srep27922)
Supplement: Supplementary Information [file srep27922-s1.pdf]

Scientific Reports

Supplementary Information

Uncovering ancient transcription systems with a novel evolutionary indicator

Naruhiko Adachi, Toshiya Senda, and Masami Horikoshi

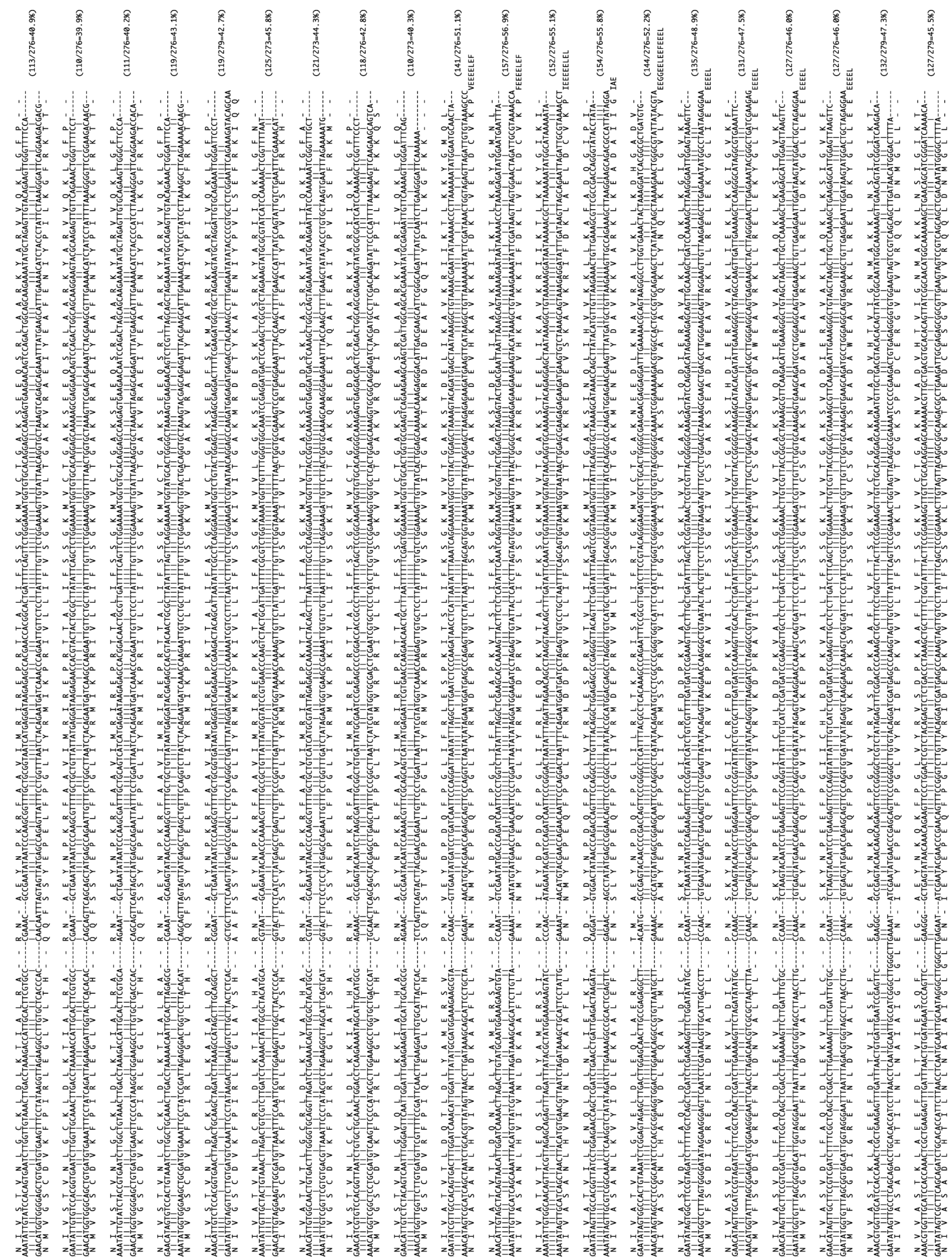

Supplementary Figure S1

(caption is shown in the next page)

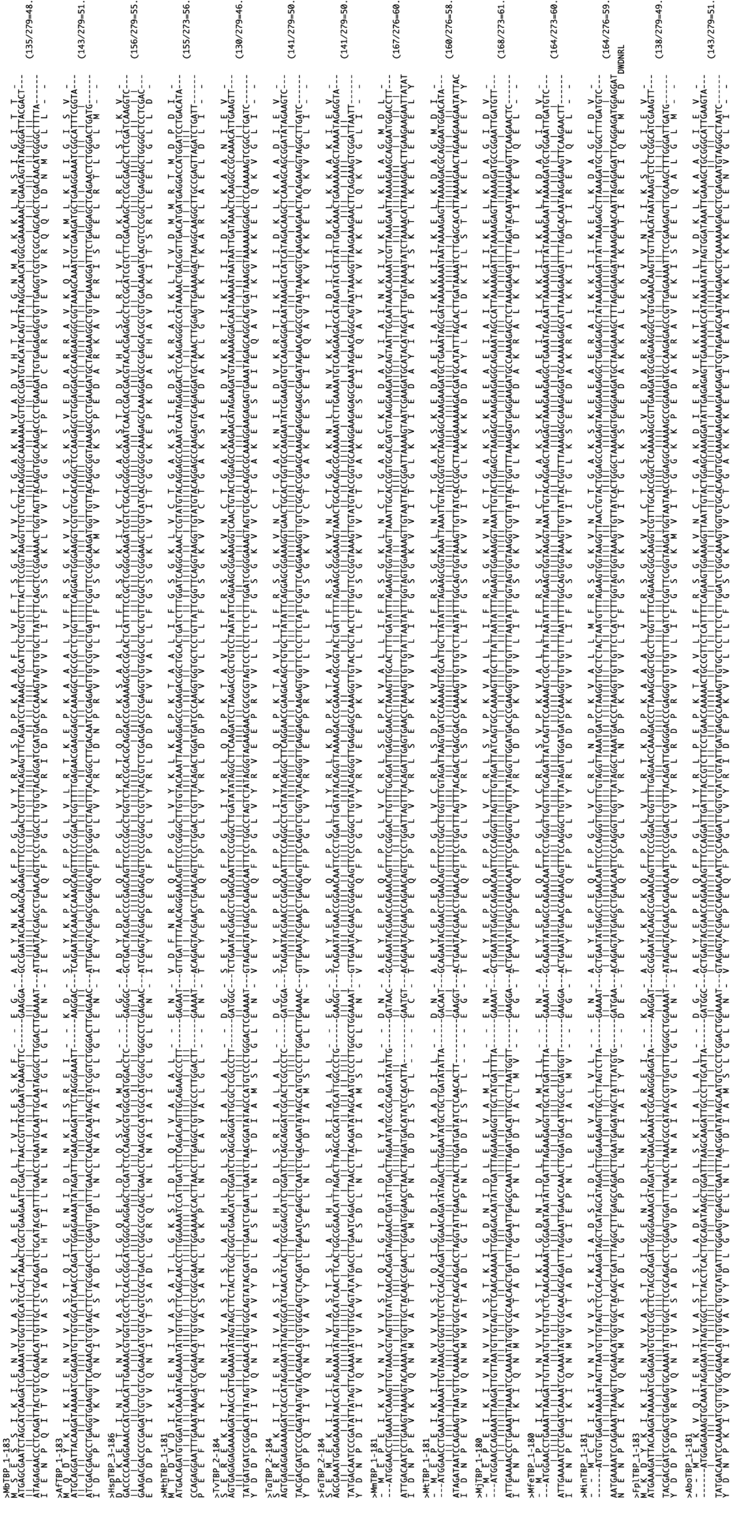

Supplementary Figure S1

Sequence alignment of direct repeats of TBP. Nucleotide sequence alignment of the conserved core region of TBP from 34 species used in this study. Identical nucleotides in the alignment are connected by vertical lines. Abbreviations of the species names are shown in the footnote of Table 1.

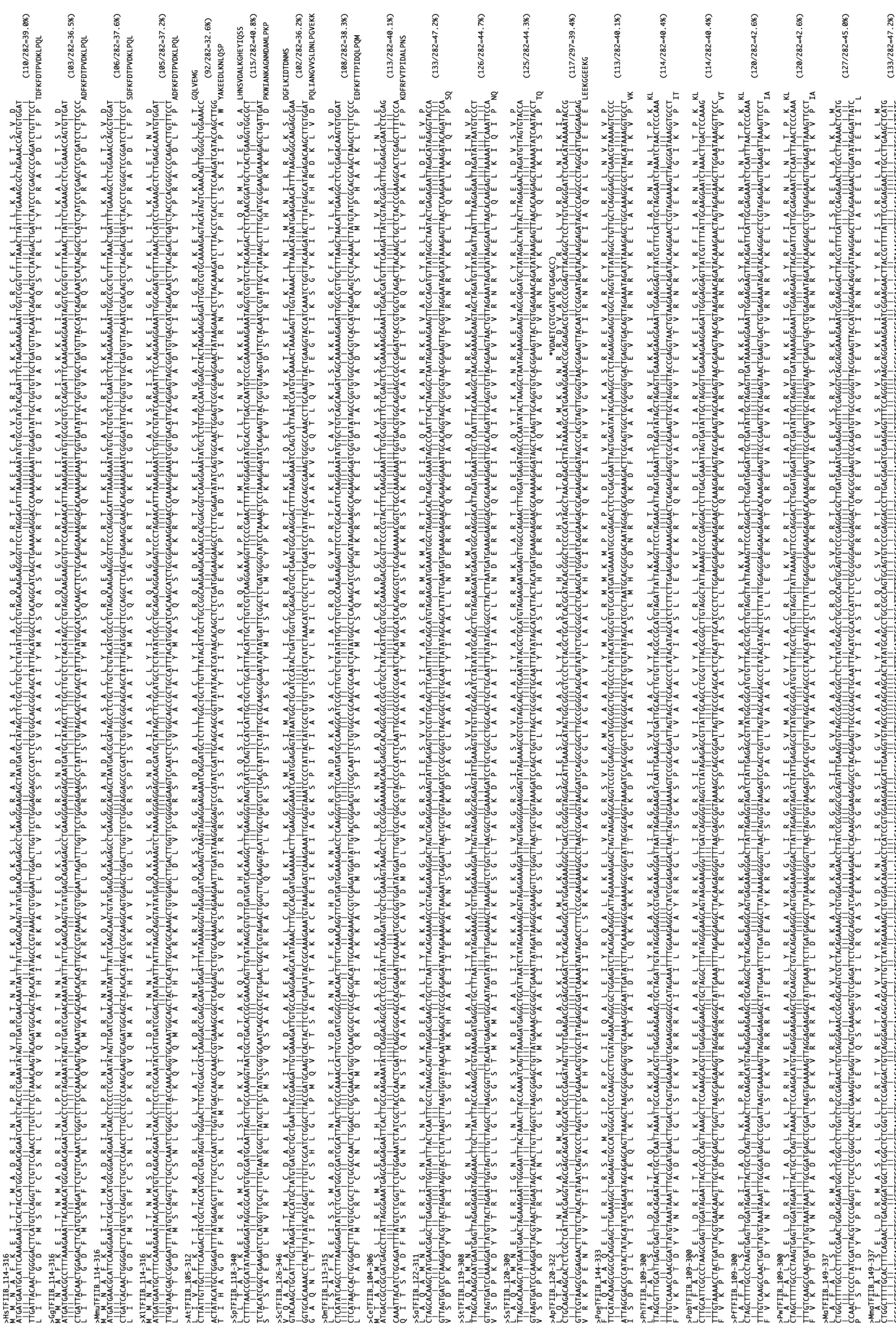

Supplementary Figure S2

(caption is shown in the next page)

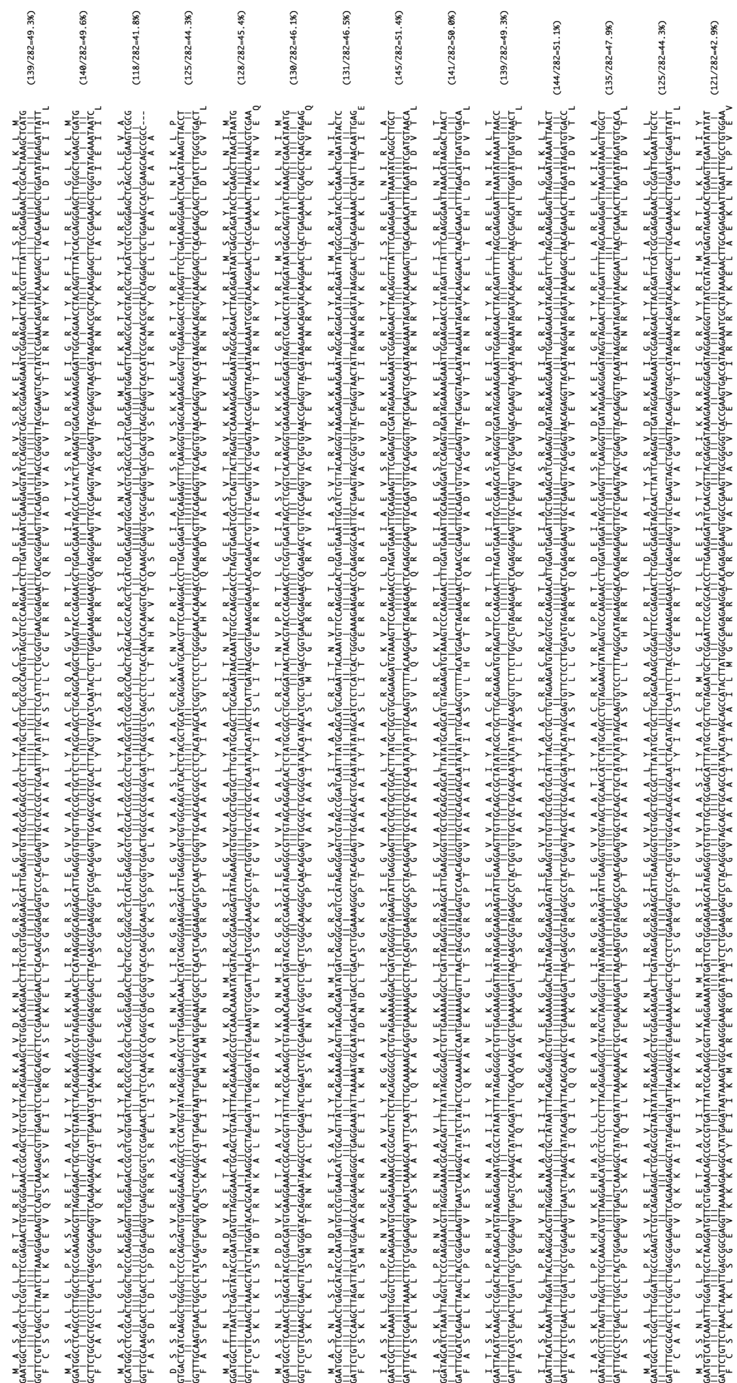

Supplementary Figure S2

Sequence alignment of direct repeats of TFIIB. Nucleotide sequence alignment of the conserved core region of TFIIB from 34 species used in this study. Identical nucleotides in the alignment are connected by vertical lines. Abbreviations of the species names are shown in the footnote of Table 1.

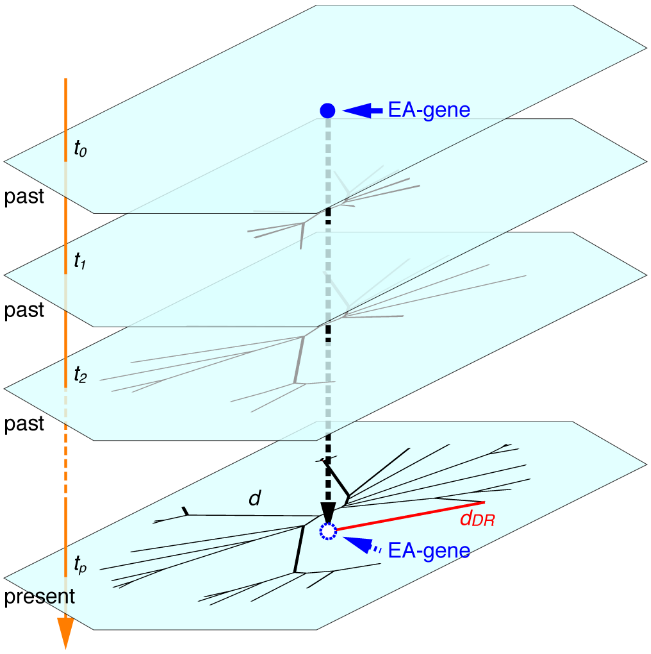

Supplementary Figure S3

A "stacked" phylogenetic tree rooted from the EA-gene, and showing the precise relationships of genes evolving through time. The blue arrow indicates an EA-gene. Each cyan plate has a phylogenetic tree (shown with gray lines) representing a particular time point in the evolution; each phylogenetic tree is prepared using gene information from various organisms living during a certain time range in the past. Since the common ancestor of TBP no longer exists in the present day (T = t_p), the phylogenetic tree at T = t_p does not have a point for the EA-gene (the projected position of the EA-gene on the plane at T = t_p is not generally located on the phylogenetic tree). It is impossible to place the position of the EA-gene on the plane at T = t_p. However, d_{DR}, which is an indicator of the evolutionary distance between a particular present offspring gene and its EA-gene, can be obtained for each gene by using direct repeat sequence information. Therefore, while the EA-gene cannot be included in the phylogenetic tree, we can obtain the information about an evolutionary distance between a particular present offspring gene and the EA-gene, which is represented by the length of the red line in the figure. The schematic drawing on the bottom plane corresponds to Figure 1F.

		amino-acid composition																			
	d_{DR}	G	A	V	L	I	M	F	W	P	S	T	N	Q	C	D	E	K	H	R	Y
Hs	1.137	11	14	15	18	15	4	11	0	9	11	9	6	7	3	3	10	15	1	14	6
Gg	1.194	11	14	15	18	15	4	11	0	9	11	9	6	7	3	3	10	15	1	14	6
Mmu	1.137	11	14	15	18	15	4	11	0	9	11	9	6	7	3	3	10	15	1	14	6
Xl	1.057	11	14	15	18	15	4	11	0	9	11	9	6	7	3	3	10	15	1	14	6
At	1.066	9	18	13	14	17	5	12	0	10	11	7	6	6	3	6	9	19	1	10	7
Sp	1.003	11	15	16	17	16	3	11	0	8	12	7	6	4	2	6	10	16	3	11	7
Sc	1.013	11	17	14	15	17	4	12	0	8	11	11	4	5	2	5	10	16	2	11	6
Dm	1.049	9	13	14	18	16	4	12	0	10	10	6	6	6	5	8	7	18	2	13	5
Ce	1.223	11	15	15	16	15	6	11	0	9	9	9	5	8	3	5	9	15	2	12	5
Sa	0.774	8	11	19	18	16	6	7	0	10	8	9	8	6	1	10	12	20	2	6	6
St	0.619	8	12	20	18	15	6	7	0	10	7	8	10	5	1	9	13	19	2	7	6
Ss	0.672	8	12	17	17	19	5	8	0	10	9	7	9	6	1	9	13	20	1	7	5
Ap	0.639	9	13	18	18	20	5	6	0	10	8	5	9	9	0	9	14	18	1	5	6
Pae	0.740	12	15	18	23	11	5	5	0	9	8	7	9	8	2	8	15	12	2	8	6
Ph	0.816	11	13	17	20	16	1	9	1	9	11	3	8	5	4	10	16	16	0	9	4
Pab	0.879	11	13	16	20	17	1	9	1	9	10	3	9	3	4	11	16	16	1	9	4
Pf	0.930	10	13	19	22	12	2	8	1	9	13	2	6	6	4	11	15	18	1	7	4
Pw	0.930	10	13	19	22	12	2	8	1	9	13	2	6	6	4	11	15	18	1	7	4
Ma	0.855	14	12	22	16	14	4	7	0	7	12	9	11	6	2	7	17	13	2	4	4
Mma	0.933	14	12	22	16	14	4	7	0	7	12	10	11	7	2	7	16	13	2	3	4
Mb	0.814	14	12	22	16	15	3	7	0	7	11	11	11	6	2	8	15	13	2	4	4
Af	0.726	14	12	21	16	16	4	4	0	8	11	5	8	6	1	9	17	17	0	10	4
Hsp	0.656	15	12	16	22	14	1	5	0	9	10	8	6	7	1	17	18	10	2	7	4
Mth	0.619	12	17	15	22	12	4	6	0	8	8	10	7	4	2	12	17	17	0	5	3
Tv	0.902	10	12	18	20	19	1	4	0	7	10	7	7	6	2	10	22	14	1	7	6
Ta	0.759	10	12	18	20	19	1	4	0	7	10	7	6	7	2	9	23	14	1	7	6
Fa	0.755	10	11	18	21	18	2	4	0	7	10	7	8	5	2	9	22	18	1	4	6
Mm	0.516	10	11	19	19	15	4	5	0	9	7	9	7	4	4	11	23	14	0	4	6
Mt	0.560	11	12	15	20	17	3	4	0	9	8	8	7	4	2	16	19	16	0	3	7
Mj	0.488	11	12	19	17	20	4	4	0	9	6	6	7	4	2	11	23	18	0	4	3
Mfe	0.524	11	12	19	17	20	4	4	0	9	6	6	7	4	2	11	23	17	0	5	3
Min	0.524	9	13	19	16	15	4	6	1	8	6	6	10	4	3	13	26	18	0	6	4
Fpl	0.737	14	15	22	16	15	3	4	0	8	8	4	8	6	1	12	14	18	0	10	5
Abo	0.699	12	13	20	22	16	2	4	0	8	6	6	7	4	2	9	20	20	0	4	6
	$r(d_{DR})$ =	0.01	0.45	−0.46	−0.21	−0.24	0.05	0.82	−0.01	0.02	0.71	0.17	−0.43	0.45	0.42	−0.77	−0.76	−0.18	0.48	0.76	0.31
	p =	0.95	0.01	0.01	0.23	0.16	0.77	0.00	0.94	0.90	0.00	0.34	0.01	0.01	0.01	0.00	0.00	0.32	0.00	0.00	0.07

Supplementary Table S1A. d_{DR} and amino-acid composition of TBP.

		amino-acid composition																			
	d_{DR}	G	A	V	L	I	M	F	W	P	S	T	N	Q	C	D	E	K	H	R	Y
Hs	1.481	7	23	11	14	17	6	7	0	8	12	10	7	8	6	9	9	13	1	15	5
Gg	1.586	8	22	11	14	17	6	7	0	8	13	9	8	8	5	9	9	13	1	15	5
Mmu	1.594	7	23	11	14	17	6	7	0	8	12	10	7	8	6	9	9	13	1	15	5
Xl	1.564	8	21	10	14	18	6	7	0	7	13	10	9	8	5	9	9	13	1	15	5
At	1.947	9	21	11	13	19	3	4	1	5	12	12	6	8	4	10	12	16	2	14	6
Sp	1.276	12	22	9	18	17	8	2	0	7	15	12	2	6	5	7	10	15	2	12	7
Sc	1.679	10	18	10	17	20	5	5	0	5	10	14	4	7	6	5	13	23	4	6	6
Dm	1.436	7	25	11	13	16	6	7	0	7	14	8	7	6	6	10	8	18	4	11	4
Ce	1.327	7	29	9	14	17	6	6	0	6	16	8	6	6	6	7	12	13	1	15	4
Sa	0.957	9	27	13	20	16	4	1	0	5	7	10	7	6	2	7	16	15	1	16	6
St	1.043	10	26	12	20	17	5	1	0	5	8	9	7	6	2	8	15	15	0	16	6
Ss	1.052	10	28	15	22	13	3	0	0	5	9	9	6	5	2	8	14	15	1	16	7
Ap	1.194	14	22	14	18	21	4	1	0	6	11	8	5	3	0	6	15	14	5	15	6
Pae	1.262	8	29	14	20	13	7	2	0	8	7	6	4	7	3	6	14	14	1	18	7
Ph	1.168	9	22	17	22	12	0	4	0	6	9	8	4	2	2	5	21	15	1	22	7
Pab	1.150	9	22	17	22	12	0	4	0	6	9	8	4	2	2	5	21	16	1	21	7
Pf	1.062	8	23	17	22	11	1	4	0	6	8	8	4	2	2	7	19	16	1	22	7
Pw	1.062	8	23	17	22	11	1	4	0	6	8	8	4	2	2	7	19	16	1	22	7
Ma	1.044	11	23	16	18	14	2	3	0	6	14	10	3	4	4	6	19	8	0	21	6
Mma	0.963	11	23	16	18	14	2	3	0	6	14	10	3	4	4	6	19	8	0	21	6
Mb	0.886	11	23	16	18	14	2	3	0	6	14	10	3	4	4	6	19	8	0	21	6
Af	0.862	14	27	14	19	15	2	3	0	6	9	11	2	3	2	4	19	11	0	20	7
Hsp	1.240	7	26	16	19	9	2	2	0	8	19	10	4	6	0	9	20	5	1	17	7
Mth	1.041	13	20	19	20	9	3	3	0	7	15	11	5	3	2	6	18	8	1	19	6
Tv	0.988	11	21	15	17	15	5	2	0	4	11	13	9	4	2	4	15	14	0	19	7
Ta	0.991	11	22	14	17	14	6	1	0	4	13	13	8	5	2	4	15	14	0	18	7
Fa	0.993	9	20	13	16	18	5	1	0	5	12	13	9	4	2	6	15	13	0	20	7
Mm	0.784	14	24	17	18	13	0	3	0	7	10	11	5	3	2	7	16	9	1	22	6
Mt	0.828	14	23	15	18	14	0	3	0	7	10	12	6	2	2	7	16	9	2	22	6
Mj	0.869	14	24	15	18	16	0	3	0	7	8	12	4	4	3	6	17	7	2	22	6
Mfe	0.820	15	24	15	19	16	0	3	0	7	8	12	3	4	3	6	17	7	2	22	6
Min	0.918	13	23	16	21	14	0	3	0	7	9	11	2	2	1	6	17	14	4	19	7
Fpl	1.035	14	28	14	19	16	1	3	0	6	9	11	2	3	2	4	19	12	0	19	7
Abo	1.100	11	19	15	15	19	6	1	0	6	10	10	5	3	2	4	19	15	0	21	8
	r(d_{DR}) =	−0.63	−0.23	−0.67	−0.60	0.43	0.54	0.61	0.50	0.18	0.31	−0.11	0.32	0.75	0.61	0.57	−0.66	0.50	0.29	−0.74	−0.44
	p =	0.00	0.18	0.00	0.00	0.01	0.00	0.00	0.00	0.31	0.08	0.54	0.07	0.00	0.00	0.00	0.00	0.00	0.10	0.00	0.01

Supplementary Table S1B. d_{DR} and amino-acid composition of TFIIB.

	(A) TBP						(B) TFIIB				
	identical bases	total	identity [%]	d_{DR}	d_{Mj}		identical bases	total	identity [%]	d_{DR}	d_{Mj}
Hs	113	276	40.9	1.14	0.828	Hs	110	282	39.0	1.48	0.954
Gg	110	276	39.9	1.19	0.830	Gg	103	282	36.5	1.59	0.894
Mmu	111	276	40.2	1.14	0.830	Mmu	106	282	37.6	1.59	1.02
Xl	119	276	43.1	1.06	0.854	Xl	105	282	37.2	1.56	0.941
At	119	279	42.7	1.07	0.842	At	92	282	32.6	1.95	1.38
Sp	125	273	45.8	1.00	0.941	Sp	115	282	40.8	1.28	1.17
Sc	121	273	44.3	1.01	0.822	Sc	102	282	36.2	1.68	1.04
Dm	118	276	42.8	1.05	0.997	Dm	108	282	38.3	1.44	1.13
Ce	110	273	40.3	1.22	0.851	Ce	113	282	40.1	1.33	1.03
Sa	141	276	51.1	0.774	0.581	Sa	133	282	47.2	0.957	0.571
St	157	276	56.9	0.619	0.535	St	126	282	44.7	1.04	0.508
Ss	152	276	55.1	0.672	0.598	Ss	125	282	44.3	1.05	0.579
Ap	154	276	55.8	0.639	0.714	Ap	117	297	39.4	1.19	0.820
Pae	144	276	52.2	0.740	0.768	Pae	113	282	40.1	1.26	0.784
Ph	135	276	48.9	0.816	0.726	Ph	114	282	40.4	1.17	0.498
Pab	131	276	47.5	0.879	0.817	Pab	114	282	40.4	1.15	0.540
Pf	127	276	46.0	0.930	0.756	Pf	120	282	42.6	1.06	0.477
Pw	127	276	46.0	0.930	0.756	Pw	120	282	42.6	1.06	0.477
Ma	132	279	47.3	0.855	0.676	Ma	127	282	45.0	1.04	0.497
Mma	127	279	45.5	0.933	0.707	Mma	133	282	47.2	0.963	0.477
Mb	135	279	48.4	0.814	0.717	Mb	139	282	49.3	0.886	0.449
Af	143	279	51.3	0.726	0.615	Af	140	282	49.6	0.862	0.485
Hsp	156	279	55.9	0.656	0.844	Hsp	118	282	41.8	1.24	1.01
Mth	155	273	56.8	0.619	0.686	Mth	125	282	44.3	1.04	0.440
Tv	130	279	46.6	0.902	0.649	Tv	128	282	45.4	0.988	0.530
Ta	141	279	50.5	0.759	0.698	Ta	130	282	46.1	0.991	0.638
Fa	141	279	50.5	0.755	0.638	Fa	131	282	46.5	0.993	0.552
Mm	167	276	60.5	0.516	0.353	Mm	145	282	51.4	0.784	0.273
Mt	160	276	58.0	0.560	0.310	Mt	141	282	50.0	0.828	0.240
Mj	168	273	61.5	0.488	0.000	Mj	139	282	49.3	0.869	0.000
Mfe	164	273	60.1	0.524	0.097	Mfe	144	282	51.1	0.820	0.154
Min	164	276	59.4	0.524	0.255	Min	135	282	47.9	0.918	0.257
Fpl	138	279	49.5	0.737	0.582	Fpl	125	282	44.3	1.04	0.419
Abo	143	279	51.3	0.699	0.580	Abo	121	282	42.9	1.10	0.565

Supplementary Table S2. d_{DR} and d_{Mj} of TBP and TFIIB.
